# Survival of Ischaemic and Haemorrhagic Stroke: Analysis of the Malaysian National Stroke Registry Data from 2009 to 2013

**DOI:** 10.21315/mjms2024.31.5.14

**Published:** 2024-10-08

**Authors:** Abd Wahab Amer Taufek, Yaacob Najib Majdi, Mohd Hairon Suhaily, Abdul Aziz Zariah

**Affiliations:** 1 Department of Community Medicine, School of Medical Sciences, Universiti Sains Malaysia Kelantan, Malaysia; 2 Biostatistics and Research Methodology Unit, School of Medical Sciences, Universiti Sains Malaysia Kelantan, Malaysia; 3 Neurology Division, Sultanah Nur Zahirah Hospital, Terengganu, Malaysia

**Keywords:** stroke, survival, Malaysia, median survival time, survival rate

## Abstract

**Background:**

Stroke ranks as the second leading cause of death globally, contributing to 15.2 million deaths in 2016. In Malaysia, stroke has emerged as a significant cause of mortality and disability. This study aims to evaluate the survival time and rate of stroke patients in Malaysia.

**Methods:**

In this retrospective cohort study, we reviewed secondary data from the National Stroke Registry (NSR) of Malaysia. The study included all Malaysian residents over the age of 12 years old diagnosed with either ischaemic or haemorrhagic stroke between 1 January 2009 and 31 December 2013. Patients with a transient ischaemic attack were excluded. We updated the death status up to 31 December 2018 using mortality data from the Malaysian National Registry Department. We used Kaplan-Meier Survival Analysis to determine the overall median survival time and log-rank test to compare the median time by ethnicity, sex and stroke type. The survival rates at 1 year, 3 years and 5 years were obtained using the life-table method.

**Results:**

The analysis included a total of 5,777 stroke patients. The mean age at diagnosis was 63.15 years old (with a standard deviation of 12.46 years old). The overall median survival time was 51 months (95% CI: 47.4, 54.6). Non-Malay patients and those with ischaemic strokes experienced a longer median survival time (65.2 months [95% CI: 56.6, 73.7] and 56.3 months [95% CI: 52.2, 60.3]), respectively. The survival rates at 1 year, 3 years and 5 years were 66.7% (95% CI: 65.5%, 68.0%), 55.6% (95% CI: 54.3%, 56.9%) and 46.9% (95% CI: 45.6%, 48.2%), respectively.

**Conclusion:**

There are significant differences in median survival time in relation to ethnicity and stroke types. Compared to other developed countries, Malaysia’s 5-year survival rate is notably lower.

## Introduction

Stroke ranks as the second leading cause of death globally, surpassed only by ischaemic heart disease and was responsible for 15.2 million deaths in 2016, marking it as a major cause of mortality worldwide over the past 15 years (1). In Malaysia, stroke ranks third as a cause of death, following ischaemic heart disease and pneumonia (2). According to the Ministry of Health Malaysia (MOH), stroke is the primary cause of death and disability among both genders (3).

The World Health Organization (WHO) defines stroke as a ‘rapidly developing clinical signs of focal (or general) disturbance of cerebral function, with symptoms lasting 24 h or more or leading to death, with no clear cause other than vascular origin’ (4). Strokes are generally categorised into three types: i) ischaemic, ii) haemorrhagic and iii) undetermined (5). This classification is also used in Malaysia, supplemented with additional stroke subtypes from the ‘trial of ORG 10172 in acute stroke treatment’ (TOAST) and Oxfordshire. According to a 2016 report by the National Stroke Registry (NSR) of Malaysia, around 76% of stroke cases in Malaysia result from ischaemic stroke (6).

The survival rate and median survival time are commonly used to report stroke outcomes. The survival rate denotes the proportion of individuals alive for a specified duration after a stroke diagnosis, while the median survival time is the time at which half of the population in a study has passed away, with the other half still alive. Stroke survival research has generated diverse findings. In studies that reviewed the 1-year survival rate, findings ranged from a low survival of 16% (7) to a high survival of 85% (8). These discrepancies are attributable to the difference in the aims and participants of each study.

Some studies report the overall median survival time among stroke patients (9–12), while others compare this statistic across varying variables of interest (13–16). The results are often dependent on differing patient characteristics and follow-up durations. A hospital-based study had been conducted in the northeast of Malaysia by Musa (17). However, this study did not represent the entire Malaysian population, as the sample only included hospital patients from this area.

To our knowledge, there is no published nationwide population-based stroke survival study in Malaysia. Therefore, the goal of this study is to determine the median survival time and the 1-, 3- and 5-year survival rates of stroke patients in Malaysia. This will fill the current knowledge gap. Additionally, identifying the median survival and prognostic factors in stroke patients will provide valuable insight for our healthcare providers. This information will aid not only active stroke treatment but also management during the recovery period. Moreover, these findings could guide policymakers in enhancing patient care, which, in turn, could improve survival rates for stroke patients.

## Methods

### Study Design

This retrospective cohort study covers the period from November 2019 to June 2020. We reviewed secondary data from the NSR of Malaysia of all stroke cases diagnosed in Malaysia between 1 January 2009 and 31 December 2013. The NSR of Malaysia, started in 2009, collects data of stroke patients from 15 hospitals across the country and is the only registry compiling all stroke cases in Malaysia. It also helps record clinical stroke data and monitor evidence-based medical practices in participating hospitals. The MOH Malaysia supports the NSR of Malaysia in improving the quality of stroke care in line with the Malaysian stroke framework.

We included Malaysian residents aged 12 years old and above diagnosed with either ischaemic stroke or haemorrhagic stroke (intracranial hemorrhage [ICH] and subarachnoid hemorrhage [SAH]) between 1 January 2009 and 31 December 2013 in this study. We excluded transient ischaemic attack (TIA) cases, foreigners and duplicated records. Per the WHO definition of stroke, we did not include TIA-diagnosed patients as the symptoms typically resolve between 1 h and 24 h (18).

The estimated sample size for determining the 1-, 3- and 5-year survival rates among stroke patients in Malaysia was calculated to be 1,869, using the sample size calculation for estimation of proportion. However, since the data is already available, we have decided to include all eligible patients (*n* = 5,777).

### Statistical Analysis

We initially acquired data for 6,272 patients from the NSR of Malaysia. Data for foreigners (*n* = 163), TIA (*n* = 132) and duplicated cases (*n* = 200) were then excluded. Following this, we retrieved information about mortality status up until 31 December 2018 from the National Registry Department. All statistical analyses were performed using the SPSS software version 24.0. Our analysis focused on time-to-event as the primary outcome. The event represented death from any cause and patients not having experienced the event labeled as ‘censored’ at the study’s conclusion on 31 December 2018. Survival time was computed in months from the date of diagnosis to either death or censoring.

We used the Kaplan-Meier survival analysis to estimate the overall survival of stroke patients and log-rank tests to compare the survival by considering variables such as ethnicity, sex and stroke type. The findings are presented as the estimated median survival time, the 95% confidence interval (CI) of median time and Kaplan-Meier survival curves. The 1-, 3- and 5-year survival rates were calculated employing the life-table method. The 5-year survival rate reflects the proportion of patients still alive 5 years post-diagnosis. These results are displayed using a life table.

## Results

In the NSR, from 2009 to 2013, there were 6,272 registered patients, of which 5,777 stroke patients were analysed. The median follow-up duration for these stroke patients was 51.02 months (IQR: 74.71). The ethnicity variable was simplified into Malay and non-Malay due to the small number of cases of other ethnicities (Chinese 10.9%, Indian 4.2%, other Bumiputera 3.6%). The patients’ mean (SD) age was 63.15 (12.46) years old. The majority of the patients were male (54.3%) and Malay (81.3%). The most prevalent type of stroke in this study was ischaemic (80.5%) and most patients did not receive stroke preventive medication (anticoagulant or antiplatelet) before the stroke event. Hypertension was present in over half of these patients and nearly half had diabetes mellitus. Characteristics of patients included in this analysis are summarised in Table 1.

### Median Survival Time

**Figure 1:**
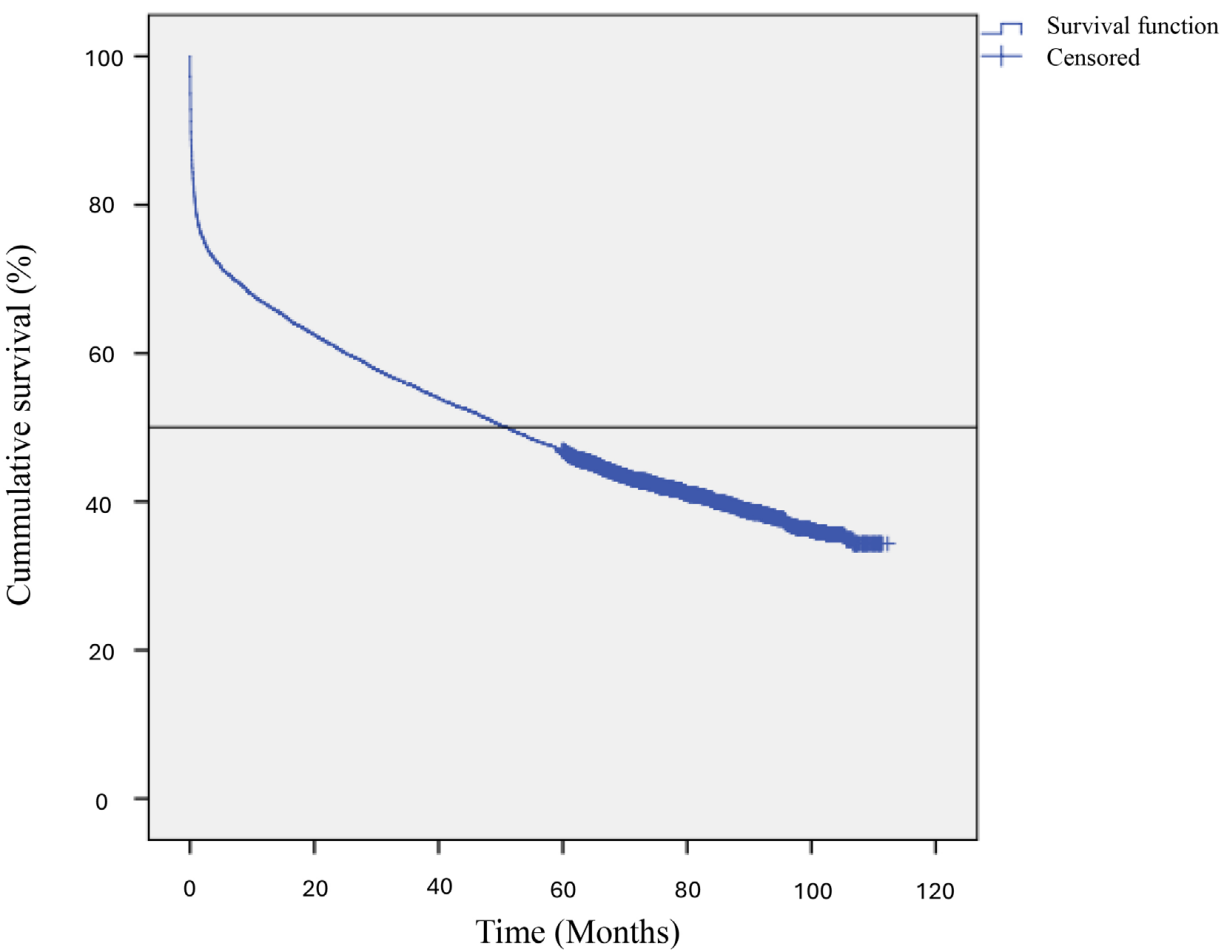
The overall Kaplan-Meier curve showing the median survival time for Malaysian stroke patients diagnosed between 2009 and 2013

The overvall median survival time for Malaysian stroke patients diagnosed between 2009 and 2013 was 51 (95% CI: 47.4, 54.6) months. The overall Kaplan-Meier curves are displayed in Figure 1. A breakdown of this median survival time by ethnicity, gender and stroke types can be viewed in Table 2.

### 1-, 3- and 5-Years Survival Rate

Five years post-diagnosis, 46.9% of overall stroke patients were still alive (95% CI: 45.6, 48.2). Table 3 outlines the 1-, 3- and 5-year stroke survival rates.

## Discussion

In our study, we explored the characteristics and survival estimates of stroke patients in Malaysia. Our results revealed that the average age at diagnosis among Malaysian stroke patients from 2009 to 2013 mirrored previously published studies (8, 13, 19). However, the mean diagnostic age was lower than that reported in Korea by Minn et al. (20), where it exceeded 80 years, yet higher than that in Estonia (21). As for gender distribution, our study found a higher prevalence in males, consistent with several global studies (7, 22, 23). Nonetheless, some research revealed a greater female prevalence (24, 25).

In Malaysia, the Malay ethnic group represents the majority of the population (26). This demographic distribution is echoed in our study, where Malays constituted the largest proportion of stroke patients at 81.3%. On the contrary, a study by Sun et al. (14) in Singapore found that Chinese people comprised the largest sample, followed by Malays and Indians. This aligns with the ethnic distribution in Singapore, which has a majority Chinese population (27). Such demographic insights are invaluable for healthcare providers and policymakers. Understanding which sections of the Malaysian population are most affected by strokes allows for improved treatment and resource distribution. By tailoring healthcare services and preventive strategies to the needs of this population, the well-being of the community can be enhanced.

Our study found that ischaemic stroke, which accounts for over two-thirds of all stroke cases, is the most common type among stroke patients. Hypertension was found in over half of the patients and roughly half had diabetes mellitus. These findings are consistent with others who also found ischaemic stroke to be the most prevalent (25, 28, 29). Our results mirror a study by Kim (8), showing SAH as the least common stroke type. We also analysed the recurrence of stroke among patients in Malaysia. Based on the data from previous studies, we found that approximately one-fifth of all stroke patients experienced recurrent strokes (12, 30). This rate is lower than the recurrence rate in Taiwan (22). These findings underscore the urgent need for effective stroke prevention programmes focusing on controlling hypertension, managing diabetes and educating patients about medication adherence. Such efforts to prevent strokes and reduce risk factors are critical not only for improving patient outcomes but also for relieving strain on healthcare systems.

In our study, the median survival time was longer compared to studies conducted in India, the United States and Denmark (9–11), suggesting better stroke treatment management in Malaysia. However, our median survival time was shorter than reported in a Sweden-based hospital study (12). Numerous studies have found a higher mortality risk associated with haemorrhagic stroke (8, 17, 30–32). This could explain the longer median survival time reported by Eriksson and Olsson (12), as their study included just a small percentage (16%) of haemorrhagic stroke cases.

Contrary to a Thai study that found the median survival time to be shorter for the ischaemic group than the haemorrhagic group (13), our study discovered that the ischaemic stroke group typically survived longer than both the ICH and SAH groups. This aligns with Sun et al.’s (14) study conducted in Singapore. Haemorrhagic strokes generally exhibit a higher mortality rate compared to ischaemic strokes (33). This disparity can be attributed to several pathophysiological processes, including vasoconstriction, blood-brain barrier disruption, an increase in microthrombi, neuroinflammation and brain oedema, among others (34, 35). These processes result in more acute and faster neurological decline compared to ischaemic strokes. The lack of neurosurgical and imaging facilities, coupled with a lack of awareness, may pose challenges in managing haemorrhagic strokes, thereby increasing their severity (36, 37).

We also analysed the median survival time among different ethnic groups in Malaysia. Notably, we observed a significant difference in median survival time between Malays and non-Malays. This finding is consistent with a study by Wolfe et al. (29) in London, which reported that Black individuals had a longer survival time compared to White individuals. These observations may be attributed to factors such as socioeconomic disparities, healthcare accessibility, cultural beliefs and genetic predispositions (38, 39). It is essential to undertake further research to identify the specific causes of this disparity. This knowledge will inform the development of targeted interventions to enhance outcomes for the Malay population.

We also investigate the 1-, 3- and 5-year survival rates of stroke patients in Malaysia. For the 1-year survival rate, our study presented a higher survival rate compared to similar research in India and the United States (9, 10), but lower in comparison to studies in Lithuania and Estonia (21, 28). The 3-year survival rate from our research exceeded that from a study in India (10) but fell short of a study in Korea (8). The 5-year survival rate in our study was lower than many published reports (9, 14, 21, 28), but superior to a study by Kostulas et al. (7) in Sweden. However, it should be noted that the study in Sweden was confined to stroke patients also suffering from diabetes. Stroke patients with underlying diabetes mellitus had a higher risk of death than stroke patients without diabetes mellitus (7, 22, 31, 40). This may affect the overall survival outcome and median survival time. Such statistics offer patients and healthcare practitioners useful insights into survival prospects following a stroke diagnosis.

Caution is advised when comparing survival rates and median survival times across stroke studies. Some studies include TIA in their samples, which might affect the overall median survival time. Notably, TIA has been reported to have no connection with the risk of death after surviving the initial stroke for at least 30 days (41).

Our study was primarily limited by the use of secondary data. This constraint was due to a high incidence of unrecorded data, particularly in stroke classification. Consequently, it hindered our ability to investigate the factors contributing to the low survival outcome. Further, because we solely used registry-provided data, it was impossible to conclusively determine whether a stroke definitively caused a death.

Our study’s main strength was its use of population-based nationwide data, which allows our findings to be generalised to the Malaysian population. In contrast, studies relying on hospital-based data could be biased due to the limited range of diseases managed, as patients receiving thorough, in-hospital treatment may not accurately represent the wider population. Furthermore, our collection of population-based data, which included updates on death statuses, also enhanced our research. This crucial information was updated for all stroke patients in the registry through the mortality registry, using unique identifiers such as personal identification numbers of Malaysian citizens, passport numbers, and numbers assigned to police and military personnel.

## Conclusion

Stroke patients in Malaysia diagnosed between 2009 and 2013 had a median survival time of 51 months. There were significant differences in this time according to ethnicity and stroke type; Malays notably had a shorter median survival time than non-Malays, and patients with SAH and ICH had shorter times compared to those with ischaemic strokes. The 1-, 3-, and 5-year survival rates were 66%, 55.6% and 46.9%, respectively. Compared to other developed nations, Malaysia’s 5-year survival rate was lower. These findings are significant for policymakers and healthcare providers in shaping tailored stroke-care and prevention strategies for the Malaysian population. To address the inequality in survival rates amongst different ethnicities, genders and stroke types, we need an approach that considers social, cultural and biological factors. Further research is also needed to understand these disparities better and to devise interventions aimed at improving stroke care and outcomes in Malaysia.

## Figures and Tables

**Table 1:** The characteristics of stroke patients in Malaysia, 2009–2013 (n = 5,777)

Variables	*n* (%)
Sociodemographic	
Age (mean, SD), years old	63.15 (12.46)
Gender	
Male	3,135 (54.3)
Female	2,642 (45.7)
Ethnic	
Malay	4,697 (81.3)
Non-Malay	1,080 (18.7)
Clinical characteristics	
WHO stroke classification	
Ischaemic	4,236 (80.5)
Intracerebral haemorrhage	942 (17.9)
Subarachnoid haemorrhage	87 (1.7)
GCS score (mean, SD)	13.06 (3.18)
NIHSS score (mean, SD)	9.14 (8.78)
Systolic blood pressure (mean, SD)	165.94 (34.86)
Diastolic blood pressure (mean, SD)	91.54 (20.31)
Blood glucose (mean, SD)	8.75 (17.06)
Recurrent episode of stroke	1,210 (20.9)
Risk factors	
Family history of stroke	378 (6.5)
Smoker	1,751 (52.1)
Hypertension	4,085 (70.7)
Diabetes mellitus	2,445 (42.3)
Ischaemic heart disease	700 (12.1)
Hyperlipidaemia	1,610 (27.9)
Atrial fibrillation	158 (2.7)
Receiving stroke prophylaxis treatment	
Antiplatelet	1,198 (20.8)
Anticoagulant	95 (1.6)

**Table 2:** The median survival time according to ethnics, gender, and types of strokes among stroke patients in Malaysia

Variables	Median survival time, months (95% CI)	*P*-value*
Sex		
Male	50.9 (46.2, 55.7)	0.588
Female	51.2 (45.7, 56.7)	
Ethnic		
Malay	47.6 (43.6, 51.6)	< 0.001
Non-Malay	65.2 (56.6, 73.7)	
Types of stroke		
Ischaemic	56.3 (52.2, 60.3)	< 0.001
Intracerebral haemorrhage	10.8 (3.7, 17.8)	
Subarachnoid haemorrhage	1.7 (0.00, 5.8)	

Note:

* Log-rank test *P*-value

**Table 3:** The 1-, 3- and 5-year stroke survival rate among stroke patients in Malaysia

Survival time	Event (*n*)	Censored (*n*)	Survival rate (%)	Lower 95% CI	Upper 95% CI
1-year	1,922	3,855	66.7	65.5	68.0
3-year	285	3,211	55.6	54.3	56.9
5-year	246	2,710	46.9	45.6	48.2
